# Infant mortality in Brazil attributable to inborn errors of metabolism associated with sudden death: a time-series study (2002–2014)

**DOI:** 10.1186/s12887-019-1421-y

**Published:** 2019-02-08

**Authors:** F. H. de Bitencourt, I. V. D. Schwartz, F. S. L. Vianna

**Affiliations:** 1 Graduate Program in Genetics and Molecular Biology, Universidade Federal do Rio Grande do Sul, Porto Alegre, Brazil; 2 Department of Genetics, Universidade Federal do Rio Grande do Sul, Porto Alegre, RS Brazil; 3 Medical Genetics Service, Hospital de Clinicas de Porto Alegre, Rua Ramiro Barcelos, 2350, Porto Alegre, RS 90035-003 Brazil; 4 Laboratório de Medicina Genômica/Laboratório de Laboratório de Pesquisa em Bioética e Ética na Ciência (LAPEBEC), Experimental Research Service, Hospital de Clínicas de Porto Alegre, Porto Alegre, Brazil

**Keywords:** Sudden death, Inborn errors of metabolism, Infant mortality

## Abstract

**Background:**

The literature suggests that 0.9 to 6% of infants who die unexpectedly may have had a metabolic disorder. At least 43 different inborn errors of metabolism (IEMs) have been associated with sudden death (SUDI). To date, the frequency of IEM-associated SUDI has not been studied in Brazil. The present study sought to characterize infant mortality related to IEMs known to cause SUDI disaggregated by each of the regions of Brazil.

**Methods:**

This was a descriptive, cross-sectional, population-based study of data obtained from the Brazilian Ministry of Health Mortality Information System (SIM). Death records were obtained for all infants (age < 1 year) who died in Brazil in 2002–2014 in whom the underlying cause of death was listed as ICD-10 codes E70 (Disorders of aromatic amino-acid metabolism), E71 (Disorders of branched-chain amino-acid metabolism and fatty-acid metabolism), E72 (Other disorders of amino-acid metabolism), or E74 (Other disorders of carbohydrate metabolism), which are known to be associated with SUDI.

**Results:**

From 2002 to 2014, 199 deaths of infants aged < 1 year were recorded in the SIM with an underlying cause corresponding to one of the IEMs of interest. The prevalence of IEM-related deaths was 0.67 per 10,000 live births (0.58–0.77). Of these 199 deaths, 18 (9.0%) occurred in the North of Brazil, 43 (21.6%) in the Northeast, 80 (40.2%) in the Southeast, 46 (23.1%) in the South, and 12 (6.0%) in the Center-West region. Across all regions of the country, ICD10-E74 was predominant.

**Conclusions:**

This 13-year time-series study provides the first analysis of the number of infant deaths in Brazil attributable to IEMs known to be associated with sudden death.

**Electronic supplementary material:**

The online version of this article (10.1186/s12887-019-1421-y) contains supplementary material, which is available to authorized users.

## Background

Inborn errors of metabolism (IEMs) are rare genetic diseases often caused by a deficient activity of a certain enzyme, which leads to partial or complete blockade of a metabolic pathway in the body and, consequently, buildup of the enzyme substrate and lack of the final product. The symptoms of IEMs vary widely, and the clinical severity of each patient depends on the metabolic pathway affected and on the accumulated or deficient metabolite [1]. Most IEMs are serious diseases associated with significant morbidity and mortality, particularly in childhood [2]. More than 700 IEMs are known to science, with a cumulative incidence of approximately 1 per 800 live births [3].

Sudden unexpected death in infancy (SUDI) is one of the most common causes of postneonatal death in the first year of life. The literature suggests that 0.9 to 6% of infants who die unexpectedly may have had a metabolic disorder [4–6]. A recent systematic review showed that at least 43 different IEMs are associated with sudden death and/or Reye’s syndrome [7].

Despite recent decline, infant mortality remains a major public health concern in Brazil. As of 2014, the infant mortality rate was 14.4 per 1,000 live births, far higher than the rates reported by countries such as Canada, Cuba, Japan, and most European nations, in which rates range from 3 to 10 per 1,000 live births [8]. To date, the frequency of IEM-associated sudden death has not been studied in Brazil.

The present study sought to characterize neonatal and infant mortality related to IEMs known to cause SUDI disaggregated by each of the regions of Brazil.

## Methods

This was a descriptive, cross-sectional, population-based study of data obtained from the Brazilian Mortality Information System of the Ministry of Health (SIM, available online at www.saude.gov.br/sim). Birth rates were obtained from the Live Births Information System (SINASC, available at http://www2.datasus.gov.br/DATASUS).

SIM is the oldest health information system in the country. Established by the Ministry of Health in 1975, it has stored nationally consolidated data since 1979. The mortality information system is universal, provides high coverage, and involves the following set of actions: a) collection of the death certificate (DC); b) cause-of-death coding; c) data processing; and d) flow and dissemination of information on deaths occurring in the country. The DC is an essential document from the legal and epidemiological standpoint, and must be completed for all deaths, including fetal deaths. In principle, responsibility for completing the DC lies with the medical doctor, as enshrined in Article 84, Chapter 10, of the Brazilian Code of Medical Ethics: “A physician may not fail to attest the death of a patient he or she had been attending to, except when there is evidence of violent death” [9].

DCs are pre-numbered consecutively and printed in triplicate by the Ministry of Health and distributed free of charge to the State Departments of Health, which will subsequently supply them to the Municipal Departments of Health for distribution to health facilities, medical examiner’s offices, death verification services, physicians, and notaries public. The disposition of each of the three copies of a DC is as follows: the first is collected by the Municipal Department of Health; the second is delivered by the decedent’s family to the office of vital records, where it will be stored for legal purposes; and the third remains in the health facility from which death was notified, to be attached to the decedent’s medical record. The DC is composed of nine blocks covering 59 variables, with one (block V) solely for recording the conditions and causes of death. It is compliant with the international death certificate template adopted by the World Health Organization (WHO) since 1948, and is particularly important as a data source for the underlying (primary) and contributing (secondary) causes of death [10]. SIM research strategy was restricted to main ICD-10 (International Statistical Classification of Diseases and Related Health Problems) categories, since is not possible stratification by subgroups or specific diseases through of this tool. In addition to that, SIM present just information recorded on DC.

The SINASC was designed by analogy with the SIM and implemented gradually by the Ministry of Health from 1990 onward. It has contained nationally consolidated data since 2004, although the degree of coverage varied during the first few years of implementation. The SINASC registry includes information on all live births in the country, with data on the pregnancy, the delivery, and the child’s condition at birth. The system’s basic document is the Live Birth Certificate [11], registration of which has been compulsory since 1999.

To collect data on IEM-related deaths, we selected all infant deaths recorded in Brazil in which the underlying cause was assigned an ICD-10 code (OMS^12^) corresponding to the list of 43 IEMs potentially associated with SUDI and/or Reye Syndrome, as described by van Rijt et al. (Additional file 1: Table S1) [7].

Death records were obtained for all infants (age < 1 year) who died in Brazil in 2002–2014 in whom the underlying cause of death was listed as ICD-10 codes E70 (Disorders of aromatic amino-acid metabolism), E71 (Disorders of branched-chain amino-acid metabolism and fatty-acid metabolism), E72 (Other disorders of amino-acid metabolism), or E74 (Other disorders of carbohydrate metabolism), which are known to be associated with sudden death. Although mitochondrial respiratory chain disorders do feature in the list, these disorders are clustered under a highly heterogeneous ICD category: E88 (Other metabolic disorders). Due to this heterogeneity and to the fact that not all diseases covered by this ICD code are associated with sudden death, we chose not to include them in analyses. The study period was established taking into account that pre-2002 data are highly incomplete, and that the most recent year for which information was available is 2014.

The underlying cause of death was defined according to the International Classification of Diseases, Sixth Version (1948), which adopted the International Form of the Medical Certificate of Cause of Death, used from 1950 to the present day. The WHO defines the underlying cause of death as “the disease or injury which initiated the train of morbid events leading directly to death, or the circumstances of the accident or violence which produced the fatal injury” [12, 13].

The frequencies of the variables of interest were calculated and used to obtain crude IEM rates, by year and location, per 1000 live births in the same area and period. Then, 95% confidence intervals were calculated for the estimated rates.

The project was approved by the Hospital de Clínicas de Porto Alegre Research Ethics Committee and by the Secretaria Municipal de Saúde de Porto Alegre Research Ehtics Committee.

## Results

**Fig. 1:**
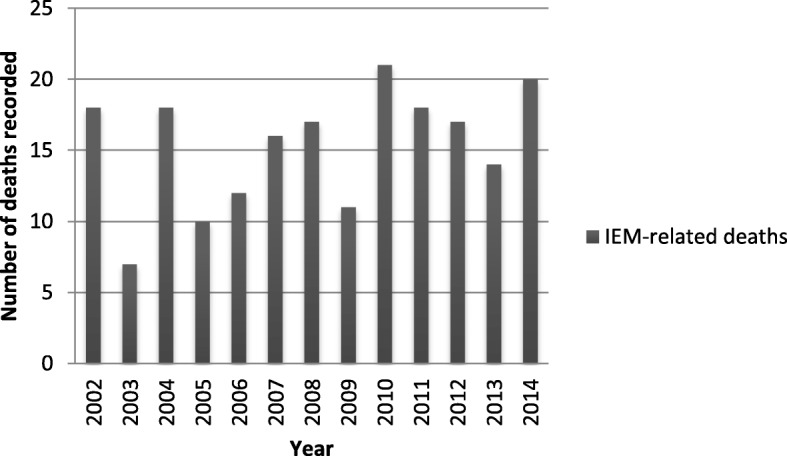
Distribution of the number of infant deaths due to IEMs recorded in Brazil, 2002–2014

From 2002 to 2014, the deaths of 598,734 children under 1 year old were recorded in Brazil. Over the same period, according to the SIM, there were 199 deaths of infants under 1 year old attributed to the IEMs of interest, which corresponds to a median 17 deaths per year (IQR: 12–18) (Fig. 1). The infant mortality rate attributable to the selected IEMs in the period of analysis was 0.67 per 10,000 live births.

Of these 199 deaths, 18 (9.0%) occurred in the North of Brazil, 43 (21.6%) in the Northeast, 80 (40.2%) in the Southeast, 46 (23.1%) in the South, and 12 (6.0%) in the Center-West region. Across all five regions of the country, ICD-10 code E74 (Other disorders of carbohydrate metabolism) was predominant; of all IEM-related infant deaths recorded in the study period, 80 (40.2%) were assigned this ICD code as the underlying cause. In the North and Southeast regions, the second leading cause was ICD-10 code E72 (Other disorders of amino-acid metabolism), whereas in the South and Northeast regions, code E70 (Disorders of aromatic amino-acid metabolism) was the second leading cause. In the Center-West region of Brazil alone, disorders classified under ICD-10 code E71 (Disorders of branched-chain amino-acid metabolism and fatty-acid metabolism) were the second leading cause of death (Table 1).

**Table 1:** Distribution of deaths due to IEMs according to ICD-10 classification, stratified by region of Brazil, 2002–2014

ICD-10	Region					
	North	Northeast	Southeast	South	Center-West	Total (%)
E70	2	12	10	11	1	36 (18.1)
E71	1	7	9	10	4	31 (15.6)
E72	8	9	26	9	0	52 (26.1)
E74	7	15	35	16	7	80 (40.2)
Total (%)	18 (9.0)	43 (21.6)	80 (40.2)	46 (23.1)	12 (6.0)	199 (100)

According to the latest demographic census at the time of writing, the population of Brazil was 202,768,562, with 2,979,259 live births in 2014 and an infant mortality rate of 14.4 per 1000. Table 2 provides infant mortality rates attributable to the IEMs of interest, using these data as a baseline.

**Table 2:** Infant mortality attributable to IEMs, stratified by region of Brazil, 2002–2014

Region of Brazil	Cases (n)	Birth per case (n)	Relative frequency (%)	Rate^a^	95% CI
South	46	396,105	23.1	1.20	0.9–1.5
Southeast	80	1,183,689	40.2	0.68	0.54–0.84
Center-West	12	245,199	6.0	0.49	2.50–0.85
Northeast	43	833,562	21.6	0.52	0.37–0.69
North	18	320,674	9.0	0.56	0.33–0.89
Brazil	199	2,979,259	100	0.67	0.58–0.77

^a^Per 10,000 births

## Discussion

According to the WHO, congenital anomalies are the second leading cause of neonatal and infant death, and they contribute to increased risk of chronic diseases and disability in many countries. Congenital anomalies, also known as birth defects, congenital disorders, or congenital malformations, can be defined as structural or functional anomalies (such as metabolic disorders) that occur during intrauterine life and can be identified prenatally, at birth or later in life. An estimated 94% of severe congenital anomalies occur in low- and middle-income countries [14]. Available at: www.who.int) [14]. Stratification of infant mortality by causes reveals that the overall mortality rate is declining in many regions worldwide, particularly that attributable to infectious causes; as a result, the proportion of such deaths attributable to congenital malformations is on the rise [15]. However, it bears stressing that structural anomalies account for the majority of congenital disorders; although metabolic derangements are considered within the definition of congenital anomalies, they are rarely reported in global statistics. Within this context, the present study was the first to evaluate infant mortality attributable to IEMs in Brazil. The data obtained show that IEM-related infant deaths may be underreported in the Center-West, North, and Northeast regions of the country, while a higher mortality rate was observed in the South.

As infectious diseases and nutrient deficiencies are being addressed, congenital and hereditary disorders are becoming increasingly pertinent in public health, and must be the object of specific official actions [16, 17].

Despite recent decline in Brazil, infant mortality remains a major public health concern. Current levels are considered high and incompatible with country development; many serious issues must be addressed to tackle this, such as persistent, notorious regional and urban inequalities [8].

In September 2000, the United Nations convened the Millennium Summit, a meeting of heads of state and government which saw the adoption of the Millennium Declaration, which sets out eight general goals to solve most of the problems faced by poor countries. Among these goals is a reduction in child mortality. In Brazil, the goal was to reduce by two thirds, by 2015, the mortality rate among children under 5. Indicators show that the infant mortality rate per 1000 live births decreased from 29.7 in 2000 to 15.6 in 2010. The most marked decline occurred in the North region, which nonetheless still has the highest rate in Brazil. The under-5 child mortality rate also declined 65% between 1990 and 2010 [18].

Disorders of beta-oxidation (included in ICD-10 code E71) appear to account for 1 to 3% of all neonatal sudden deaths [19–21]. A study by Dott and colleagues (2006) showed that the contribution of fatty acid disorders and organic acidemias in cases of SUDI in children under 3 years old is about 1% [22]. The methodology used was post-mortem tandem spectrometry (previously reported by Chace and colleagues, 2001) [5]. Fatty-oxidation disorders are associated with hypoglycemia and metabolic crisis, which can cause sudden death, as a consequence of the privation of the use of fat or protein as an alternative energy source during of fasting and/or increased metabolic demand [23].

Contradicting reports in the literature, we found that ICD-10 code E71 was least prevalent as a cause of death. This may be associated with the fact that the complexity involved in diagnosis of these diseases, combined with a lack of expertise and resources for metabolic investigation in SUDI cases, leads to under-investigation and underdiagnosis [24]. Furthermore, metabolic autopsy is not performed in cases of sudden death in Brazil.

Neonatal screening, also known as the heel-stick test, is a preventive action designed to diagnose a variety of neonatal and infectious diseases which are asymptomatic in the neonatal period, thus allowing early intervention and disease modification through specific treatment to mitigate or altogether prevent any associated clinical sequelae. Neonatal screening has been mandatory throughout Brazil since the 1990s. In 2001, the Brazilian Ministry of Health implemented the National Neonatal Screening Program, seeking to expand existing screening opportunities and include early detection of other congenital diseases. The conditions currently included are phenylketonuria, congenital hypothyroidism, sickle-cell disease, hemoglobinopathies, cystic fibrosis, congenital adrenal hyperplasia, and biotinidase deficiency. It’s important to notice that in Brazil, the Neonatal Screening it’s not made by tandem mass spectrometry [25].

A review of the literature conducted by van Rijt et al. shows that at least 43 IEMs are associated with SUDI and/or Reye Syndrome, 26 of which can cause symptoms as early as the neonatal period. At least 32 of these IEMs are treatable, and 26 can be detected by tandem mass spectrometry screening [7]. Of the IEMs associated with sudden death according to van Rijt et al., only biotinidase deficiency (ICD-10 E71) is part of the Brazilian neonatal screening program and it was included in it just in 2013 (with universal access in the whole country only in 2014) [7, 26]. Besides the late inclusion of biotinidase deficiency in the screening program, we found that ICD-10 code E71 was the least prevalent cause of IEM-related infant death. Inclusion of this disease in the neonatal screening program probably leads to early diagnosis and, consequently, rapid initiation of appropriate treatment, thereby reducing mortality.

The isolated incidence of each of the IEMs of interest was very small, which is consistent with the fact that most are inherited in an autosomal recessive pattern. However, the cumulative incidence of all IEMs is approximately 1 in 800 live births [3]. The small number of IEM-related deaths recorded in the period of analysis (199 cases in 13 years; 0.67 deaths per 10,000 live births) may represent not the rarity of the underlying disorders, but rather their underdiagnosis. Failure to enter a death into vital records, whether due to difficulty in doing so, lack of guidance, burial in irregular cemeteries, or simple lack of knowledge of the importance of death certificates among the population makes it difficult to measure the true magnitude of the problem and identify health interventions that might reduce mortality rates [27].It is important to highlight that Brazil is politically and geographically divided into five regions: North, Northeast, Southeast, South, and Center-West, each of which has distinct physical, demographic, and socioeconomic characteristics. The Southeast is the most populated region, while the Center-West is least populated.

The low information quality of DCs, represented by a large contingent of poorly defined or imprecise causes of death—so-called “junk codes”—and unfilled fields, hinders analysis of the factors that contribute to mortality and, consequently, makes it difficult to implement interventions [27]. A 2010 Brazilian study showed that physicians often found it difficult to establish the underlying cause of death, an essential piece of information that allows SIM coding. In the same study, 68% of respondents reported general difficulty in completing DCs. The large number of fields in the document and the lack of information on the patient were also reported as factors that hinder DC completion [28]. This low quality of death registration may be an additional possible cause for the low rate of IEM-related deaths during the study period. Furthermore, the growing investment in and improvement of the SIM notwithstanding, underreporting of death is still a significant issue, especially in North and Northeast Brazil [29].In 2013, the Office of the General Coordination for Epidemiological Analyses published the first and only document consolidating SIM data for the period 2005–2011. According to this publication, the SIM coverage rate—defined as the ratio of deaths recorded in SIM to the number of deaths predicted by the Brazilian Institute of Geography and Statistics—was 96.1%. Coverage approached 100% in nearly all states in the South, Southeast and Center-West regions. In the North and Northeast regions, some states reported > 90% coverage, while others still had rates in the 80–90% range [30]. Underreporting of events and the high rate of poorly defined causes of death (approximately 7.0%), in addition to improperly completed or incomplete DCs, lead to variation in the quality of available mortality data [30–32].

According to the Brazilian Society of Medical Genetics and Horovitz et al., the Southeast and South regions of the country also have the largest number of specialized medical genetics centers [33]. Most of these facilities are located in the Southeast region, particularly in the state of São Paulo. In the South region, clinical and laboratory coverage is available across all three states. Except in the state of São Paulo, the vast majority of medical genetics centers in Brazil are located in state capitals [16]. This geographical distribution of specialized centers may be associated with a greater number of diagnoses and, consequently, of reported deaths in the Southeast and South regions. Furthermore, considering that the Southeast region has the highest rate of live births in the country, it would be expected to account for a larger number of deaths overall and, consequently, of IEM-related deaths.Consanguinity increases the prevalence of congenital rare diseases and approximately doubles the risk of neonatal and infant death [14]. Bronberg et al. established the rate and spatial distribution of consanguinity in South America through analysis of information from around 127,000 live births of infants without congenital malformations delivered at hospitals affiliated with the ECLAMC (Latin American Collaborative Study of Congenital Malformations) from 1967 to 2011. Their results show that Brazil has one cluster of high consanguinity rates (1.59%) in the Southeast region of the country; and two clusters of medium consanguinity rates (0.76 and 1.22%) in the Northeast and South regions, respectively [34]. Another study reported finding several genetic isolates in different cities across the Southeast region, such as spinocerebellar ataxia type 1 in São Paulo and spinocerebellar ataxia type 3 in Rio de Janeiro [35]. These data corroborate the findings of the present study, in which the highest IEM-related infant mortality rates during the period of analysis were reported in the South and Southeast regions. However, it bears stressing that most published studies on consanguinity in Brazil have focused precisely on the South and Southeast regions of the country.

Although studies have shown very high rates of consanguinity in rural areas in the Northeast region (6 to 41%) [36, 37], our study detected underdiagnosis of IEMs in this region, as the proportion of IEM-related deaths recorded during the study period was lower than the proportion of live births in the region and the regional IEM mortality rate was lower than the overall countrywide rate. One plausible explanation for this finding is that, despite growing investment in and improvement of the SIM, underreporting of death is still a significant issue in North and Northeast Brazil [29].Likewise, our findings suggest that IEMs are underdiagnosed in the Center-West region of Brazil as well. According to the Brazilian Society of Medical Genetics, there are only seven specialized medical genetics centers across the entire region, two of which operate exclusively in the field of oncology [33]. Possibly, the smaller number of records from this region may be due to the scarcity of specialized centers, which may hinder access to diagnosis.

Some particular difficulties related to the study of IEM-related infant mortality in developing countries must be mentioned. First, there is the difficulty of classifying IEMs within the ICD-10 framework. Diseases associated with sudden death, such as mitochondrial chain disorders, are classified under highly heterogeneous categories that include different IEM groups. Another point to consider in Brazil is the SIM search function. The search strategy is restricted to main ICD-10 categories, and does not allow stratification by subgroups. For instance, although tyrosinemia corresponds to ICD-10 code E70.2 (Disorders of tyrosine metabolism) and classical phenylketonuria to ICD-10 code E70.0, the SIM would only allow searching for code E70. As SIM searches are excessively broad, we may have included deaths in our sample that were not necessarily caused by IEMs known to be associated with sudden death.

One point that should be highlighted is that the data that feed SIM are not integrated with other health systems (such as SINASC) and a great number of information cannot be recovered. It not be possible to obtain and to cross information related to the birth or notifiable diseases that a particular individual contracted during the life, in order to relate to cause of death [38]. It means that we didn’t have access to information about diagnosis (how was the diagnosis achieved in every case – based on biochemical markers, enzymatic activity, genetic tests, or others – and if the diagnosis occurred before or after death), if children were on treatment for IEM or under any medical control.

Another relevant issue is that autopsy is not always available or performed. When available, as in Brazil (during the study period, autopsy was mandatory for all infants who died at home), it is usually performed by a general pathologist and does not include microscopic studies and tests geared specifically to diagnosis of IEM, which may explain the underdiagnosis of IEMs as a primary cause of death in the assessed cases. Furthermore, lack of knowledge and limited training of medical practitioners in completion of death certificates may contribute to under-registration of IEM-related deaths [27]. In addition, although Brazilian Ministry of Health Ordinance No. 199 established the National Policy for Comprehensive Care of Persons with Rare Diseases, neither expanded neonatal screening (which would allow early diagnosis of some IEMs) nor diagnostic confirmation of such disorders are available through the unified health system [39].

The limitations of this study notwithstanding, it should be noted that SUDI remains a major cause of infant mortality, and the present investigation was the first to evaluate infant mortality caused by IEMs known to be associated with sudden death. This article also provides a comprehensive panorama of the last 13 years of operation of the SIM, an essential tool for collection of mortality data recorded in Brazil.

## Conclusions

This was the first study to assess the relationship between sudden infant death and IEMs in Brazil. The low death rate observed is thought to denote not only the rarity of these conditions, but rather underreporting. Studies of infant mortality are essential for health surveillance activities and to support decision-making by health managers, and serve as essential inputs for the public policy-making process and to assess the outcomes and impacts of such policies.

This 13-year time-series study provides the first analysis of the number of infant deaths in Brazil attributable to IEMs known to be associated with sudden death. Underreporting may be associated with the scarcity of specialized medical genetics centers, as well as to insufficient training of health providers in proper completion of death certificates. There is a clear unmet need for strategies targeting the incidence of IEMs, which should allow not only estimation of the true impact of these disorders on infant mortality but also development of prevention strategies.

## Additional file

Additional file 1: **Table S1.** Inborn errors of metabolism associated with sudden death. After van Rijt [7]. (DOCX 15 kb)

## Abbreviations

DC: Death certificate
ICD: International Statistical Classification of Diseases and Related Health Problems
IEM: Inborn errors of metabolism
SIM: Brazilian Mortality Information System of the Ministry of Health (Sistema de Informações sobre Mortalidade)
SINASC: Live Births Information System (Sistema de Informações sobre Nascidos Vivos)
SUDI: Sudden unexpected death in infancy
WHO: World Health Organization
