# Supplementary material for: Infant mortality in Brazil attributable to inborn errors of metabolism associated with sudden death: a time-series study (2002–2014)
Source: BMC Pediatr. 2019 Feb 8;19:52. doi: 10.1186/s12887-019-1421-y (PMC6367785; doi:10.1186/s12887-019-1421-y)
Supplement: Supplementary file 1 — Table S1. Inborn errors of metabolism associated with sudden death. After van Rijt [7]. (DOCX 15 kb) [file 12887_2019_1421_MOESM1_ESM.docx]

**Additional file 1: Table S1.** Inborn errors of metabolism associated with sudden death. After van Rijt [7].

| **Metabolic diseases associated with sudden death** | **ICD-10** |
| --- | --- |
| *Amino-acid and peptide metabolism*  Urea cycle disorders  Carbamoylphosphate synthetase deficiency  Ornithine transcarbamylase deficiency  Citrullinemia type I  Argininosuccinic aciduria  Organic acidemias  Glutaric acidemia type I  Methylmalonic acidemia  Isovaleric acidemia  Methylglutaconic acidemia type I  L-2-hydroxyglutaric acidemia  Disorders of biotin metabolism  Biotinidase deficiency  Disorders of phenylalanine or tyrosine metabolism  Tyrosinemia type I  Disorders of glycine or serine metabolism  Nonketotic hyperglycinemia  Disorders of amino-acid transport  Lysinuric protein intolerance | E72  E71  E71  E70  E72  E72 |
| *Carbohydrate metabolism*  Disorders of gluconeogenesis  Phosphoenolpyruvate carboxykinase deficiency  Glycogen storage disorders  Glycogen storage disease type Ia  Glycogen storage disease type Ib  Glycogen storage disease type II | E74  E74 |
| *Fatty acid and ketone body metabolism*  Disorders of carnitine transport and the carnitine cycle  Carnitine transporter deficiency  Carnitine palmitoyltransferase I deficiency  Carnitine-acylcarnitine translocase deficiency  Carnitine palmitoyltransferase II deficiency  Disorders of mitochondrial fatty acid oxidation  Very long-chain acyl-CoA dehydrogenase deficiency  Medium-chain acyl-CoA dehydrogenase deficiency  Multiple acyl-CoA dehydrogenase deficiency | E71  E71 |
| *Energy metabolism*  Mitochondrial respiratory chain disorders | E88 |
